# Daily sound exposure of hearing aids users during COVID-19 pandemic in Europe

**DOI:** 10.3389/fpubh.2023.1091706

**Published:** 2023-10-13

**Authors:** Kang Sun, Tiberiu-Ioan Szatmari, Alessandro Pasta, Lars Bramsløw, Dorothea Wendt, Jeppe H. Christensen, Niels H. Pontoppidan

**Affiliations:** ^1^Eriksholm Research Centre, Snekkersten, Denmark; ^2^Department of Applied Mathematics and Computer Science, Technical University of Denmark, Kongens Lyngby, Denmark; ^3^Demant A/S, Smørum, Denmark; ^4^Hearing Systems, Department of Health Technology, Technical University of Denmark, Kongens Lyngby, Denmark

**Keywords:** COVID-19, hearing aid users, governance intervention, sound exposure, sound pressure level, signal-to-noise ratio

## Abstract

**Introduction:**

This study aimed to investigate the daily sound exposure of hearing aid (HA) users during the COVID-19 pandemic, with a specific focus on the impact of different governance intervention levels.

**Methods:**

Modern HA technology was employed to measure and compare the sound exposure of HA users in three distinct periods: pre-pandemic, and two 14-day periods during the pandemic, corresponding to varying levels of governance interventions. The study sample comprised a total of 386 HA users in Europe during the pandemic, with daily sound exposure data collected as part of the main dataset.

**Results:**

The results revealed that, during the pandemic, the equivalent continuous sound pressure level (SPL) experienced by HA users decreased, while the signal-to-noise ratio (SNR) increased compared to the pre-pandemic period. Notably, this impact was found to be more pronounced (*p* < 0.05) when individuals were subjected to stronger governance intervention levels, characterized by lower SPL and higher SNR.

**Discussion:**

This study highlights the changes in daily sound exposure experienced by HA users during the COVID-19 pandemic, particularly influenced by the extent of governance interventions that restricted social activities. These findings emphasize the importance of considering the effects of pandemic-related governance measures on the sound environments of HA users and have implications for audiological interventions and support strategies during similar crises.

## 1. Introduction

Globally more than 1.5 billion people experience some decline in their hearing capacity during their life course, of whom at least 430 million will require care (1). Hearing-impaired (HI) adults have different lifestyle and behaviors compared to normal hearing (NH) persons (2–4). In particular, HI individuals are more likely to experience emotional distress and social engagement restrictions than NH (5), and adopt more “control” and “avoidance” strategies in social situations (2). Hearing loss (HL), which is common in older adults (6), was suspected as an important risk factor for limited social engagement (7). Since limited social engagement is characterized by infrequent social interactions and participations in social activities (8), it may be linked to lower sound exposure in daily life compared to NH individuals. That is, environmental sound exposure could serve as a good measure of the behavioral difference between HI and NH. However, typical noise exposures in contemporary daily life are not well known (9), and there is a lack of research focusing on the sound exposure of HI in a less controlled daily life context (10).

The global spread of the 2019 novel coronavirus (COVID-19) has been characterized as a pandemic by the World Health Organization since March 11, 2020 (11). The on-going COVID-19 pandemic has led governments to implement various social distancing laws or recommendations, such as closedown, lockdown, closing borders, etc. While many studies focused on the effect of these measures on mitigating the spread of COVID-19 (12, 13), several recent studies have taken advantage of this reduced human activity, which implies a reduction in anthropogenic emissions, to evaluate the environmental impact associated with such interventions. Some studies (14, 15) reported that the reduced human mobility during the COVID-19 pandemic (partially or completely) mediated various indicators of air quality. Braga et al. (16) found unprecedented water transparency due to reduced human activities (e.g., boat traffic and tourism) in the Venice Lagoon. More evidence (17, 18) pointed out its positive impact on the environment. A few studies have reported the impact of the imposed restrictions on noise emissions in the urban context. Rumpler et al. (19, 20) reported up to 4 dBA urban noise level reduction during the “first wave” and a gradual lower reduction (around 1 dBA) due to lower policy compliance in the “second wave” in Stockholm, Sweden. Xiao et al. (21) analyzed seismic noise with frequencies above 1 Hz (identified to be primarily generated by local transportation systems) impacted by the restrictions in China and Italy. Their result revealed that it led to a large range of noise decrease (1–12 dB) across different monitoring sites.

Governance interventions during the pandemic were intended to interrupt the spread of the virus, but they inevitably brought adverse effects such as increased mental disorders, including anxiety and depression (22), and health problems such as insomnia, denial, anger, and fear (23). These adverse health effects may be linked to limited social interactions, which could potentially manifest in the form of sound exposure. Therefore, it is important to understand the sound exposure of HI during the COVID-19 pandemic, which was the first objective of this study. Fortunately, with the rapid development of hearing aids (HA) in the past decades (24, 25), they can now act as sound exposure monitoring devices (26). In Europe, 11.1% of the population or 58.5 million people self-reporting hearing loss, among which 33% use hearing aids (27). The daily sound exposure of HA users has recently been explored. One of the most pronounced examples, EVOTION,^1^ has illustrated the quantitively sound data collection process in their first outcome (28).

**Figure 1:**
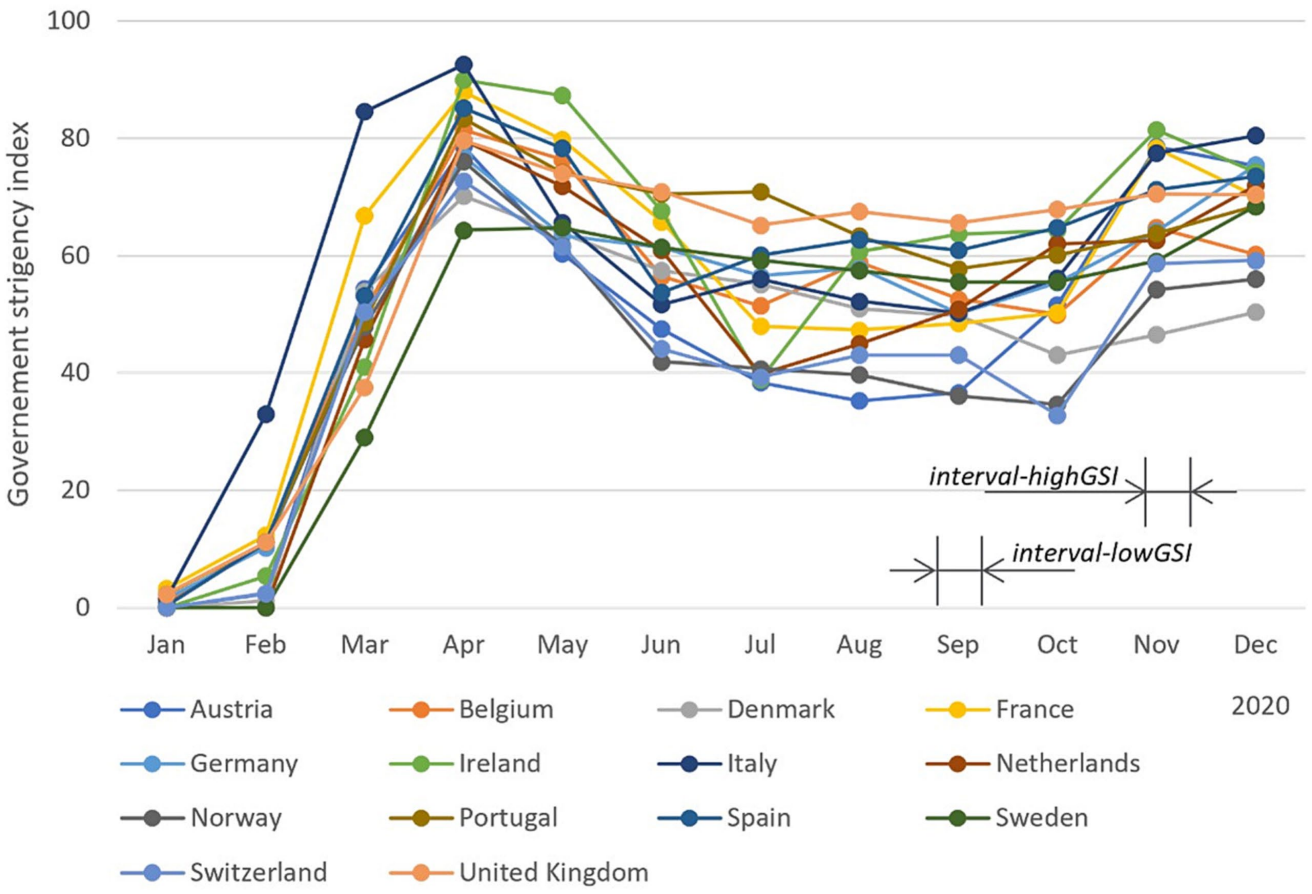
Month-based government stringency index (GSI) in European countries across 2020. This index records the number and strictness of government policies and should not be interpreted as direct “scoring” the appropriateness or effectiveness of a country’s response. The GSI data were obtained from Hale et al. (29). For day-based government stringency index and other details, we refer readers to the original source.

Government interventions in response to the COVID-19 pandemic varied widely across European countries throughout 2020. Although there were differences in terms of restriction level, effect time, affected population, a similar overall trend could be observed. Figure 1 displays the government stringency index (GSI) for European countries on a month-by-month basis throughout 2020 [by compiling data from (29)], which provides a summary of the various restriction policies implemented. The stringency index is further detailed in the Appendix. Figure 1 shows a clear “first wave” and “second wave” in the GSI, respectively in April and November.

As the intervention strength fluctuated, the sound exposure of individuals could have also been affected. For example, Redel-Macías et al. (30) conducted an online survey and analyzed voluntarily submitted sound recordings across Spain, which revealed an average reduction of over 10 dB for daytime (*L*_d_) and evening (*L*_e_) noise level during the lockdown, along with surprisingly better-perceived sound quality. However, the overall noise level increased as the restrictions were relaxed. Therefore, the second objective of this study was to investigate whether the sound exposure of HA users during the COVID-19 pandemic changed due to changes in the governance intervention strength.

With the above in mind, this study aimed to monitor environmental sound exposure of HA users during the COVID-19 pandemic, particularly under varying levels of governance interventions. To achieve this goal, the study utilized HearingFitness^™^ (31), a platform that allows for the voluntary submissions of acoustic environment information from HA users. Acoustic variables collected in this study include sound pressure level (SPL) and signal-to-noise ratio (SNR). Additionally, this study focused on HA users residing in European countries.

The study selected different time intervals (marked in Figure 1) to examine the difference in sound exposure under relatively constant governance interventions. Each interval lasted for 14 days and was referred to as an *interval* in this study. It is worth noting that the interpretation of the stringency index has been challenged, as the direct comparison between countries can be problematic. Therefore, instead of using the stringency index as a numerical factor, two periods with rather different stringency levels were selected for comparison (32, 33). The results compared the aggregated equivalent sound exposure between pre-pandemic and in-pandemic periods across the course of a day, as well as between under high- and low-level GSI.

The primary hypothesis of this study is whether there is a significant change in the sound exposure of HA users during the COVID-19 pandemic as a result of changes in environmental factors due to governance interventions. In other words, we investigate whether a change in environmental factors due to governance interventions also leads to a change in the sound exposure of HA users. The findings can contribute to our understanding of how modifications in societal conditions can impact the behavior of populations with HL and, consequently, necessitate clinical protocol adaptations.

## 2. Methodology

### 2.1. Participants and ethics

The study included participants who were users of Oticon Opn hearing aids (Oticon A/S, Smørum, Denmark) in Europe and had signed up for the HearingFitness^™^ feature (31, 34) through the Oticon ON^™^ remote control app. When signing up, participants provided consent for their anonymized data (i.e., no personal identifiers were available) to be used for research purposes at aggregated levels (i.e., no single-case investigations are performed) and agreed that data could be stored on secure servers owned by Oticon A/S (35). No demographic information including age, gender, audiogram, and exact location (GPS), was collected to ensure participant privacy. Data collection and storage followed the principles of “privacy by design” in accordance with the General Data Protection Regulation (EU regulation 2016/679). No ethical approval was necessary for this study according to the Danish National Scientific Ethical Committee.^2^ It should be emphasized that HearingFitness^™^ is commercially available beyond the scope of this study. The data utilized in this study was acquired from the secure server owned by Oticon using date and time zone filter (details see below).

The study focused on participants from European countries and compared their sound exposure with GSI. Since no geographic location information was recorded, an alternative approach was adopted to separate European participants from non-European participants based on their time zone. This approach was inspired by earlier research such as Tweet’s determination (36). This study included participants with time zones UTC, UTC + 01, UTC + 02, and UTC + 03 (37), in which participants were primarily from Europe.

### 2.2. Data

#### 2.2.1. General information

Participants who signed up for HearingFitness^™^ and had a stable Bluetooth connection between their HA and the Oticon ON^™^ app on their smartphones enabled uploading time-zone-specific timestamped HA usage data, which were registered every 10 min. The reliability of the smartphone-generated time zone data was ensured due to the requirement of a stable internet connection. Hereafter, the single occurrence of data entering the server is referred to as a submission. Each submission contained timestamped sound data that described the ambient environment using two acoustic variables.

#### 2.2.2. Time interval selection

To measure sound exposure under different government COVID-19 intervention levels, two observation intervals were selected. The first interval, referred to as “*interval-highGSI*,” started from Monday, November 2, 2020 and lasted for 14 days (until Sunday, November 15), a period during which many European countries had taken stronger interventions (higher GSI). The second interval, referred to as “*interval-lowGSI*,” was selected to measure sound exposure under a relatively relaxed governance intervention (lower GSI), and started on Monday, September 7, 2020 and ended on Sunday, September 20, 2020. The length of these two intervals was set as 14 days each to have a relatively long observation to exclude odd single observations within the observation period, and to avoid fluctuations in the GSI. The two intervals were chosen close in time to ensure that a reasonable number of participants provided data in both intervals.

Furthermore, to compare the sound exposure during *interval-lowGSI* and *interval-highGSI* with the one before the pandemic, an interval from September 1, 2019 to November 30, 2019 was selected. This interval is labeled as “*interval-pre*” hereafter. Note that in the ideal scenario, we would have had the same participants and the same time interval for both the pre-pandemic and in-pandemic periods, with just a one-year difference. Unfortunately, the limited data availability in pre-pandemic days made it not feasible in this study. Additionally, there was no overlap in participants between pre-pandemic and in-pandemic due to the significant time gap.

In “Final data description,” we provided an overview of the dataset analyzed in this study, and systematically described the selected intervals.

#### 2.2.3. Sound data

The smartphone-connected HA logged timestamped sound data describing the ambient acoustic conditions. The continuous data represent the acoustic characteristics of the momentary sound wave sensed by calibrated HA microphones at the ear level. The data for the study consists of two acoustic variables: SPL and SNR. These variables are both estimated in a broadband frequency range (0–10 kHz) in decibel units. SPL is the level output estimate from a low-pass infinite impulse response filter with a time constant of 63 ms. Signal-to-noise ratio is the difference between the bottom tracker (implemented with a slow dynamic attack time of 1 to 5 s and a fast release time of 30 ms) and the immediate SPL. Thus, SPL and SNR values exhibit dynamic changes on the same time scale. Data on the acoustic environment are logged every 10 min, including timestamps indicating the activation of the hearing aids and their connection to a smartphone via Bluetooth.

To clarify, the acoustic measures represent the intensity of the sound environment detected by the HA microphones and do not account for the amplification provided by the HA. For more detailed technical information regarding the sound level readings of the device, please refer to the Oticon website^3^ under the Technical Datasheet section in Download Center.

The sound data was organized based on the hour it was submitted on any given day. If a participant submitted more than one submission within the same hour, the data was aggregated using the logarithmic average (i.e., equivalent continuous SPL and SNR). This procedure was first applied to submissions from a single participant, and then across participants to calculate the grand average. Additionally, the number of participants having submitted data during any given hour was recorded.

#### 2.2.4. Data cleaning

To avoid the impact of spuriously extreme sound environment readings, submissions with a value greater than 110 dB SPL or 80 dB SNR (or both) were excluded, resulting in removal of approximately 5% of submissions from each dataset, respectively. Note that the data deletion adopted a listwise approach, meaning that in a single submission, if one acoustic variable exceeded the criteria in a single submission, this submission was dropped, even if the other acoustic variable was within the criteria. Moreover, a minimum of 10 submissions was required in each interval for each participant, resulting in excluding 29 participants from D2020 but none in D2019 (see “Final data description” for final numbers). This was to avoid a single or small number of submissions representing an entire interval.

#### 2.2.5. Final data description

Table 1 summarizes the datasets used in this study in participants and intervals. Data in 2020 (D2020) contained 386 participants, who submitted data in both *interval-highGSI* and *interval-lowGSI*. Data in 2019 (D2019) comprised 13 participants observed in *interval-pre*, which was acquired from Christensen et al. (38). Note that the submissions in *interval-highGSI* and *interval-lowGSI* spanned across all 24 h, while the submissions in *interval-pre* covered from 6 to 12 am. None of the 13 participants in D2019 appeared in D2020. Furthermore, in D2020, the participant distribution across different time zones (UTC, UTC + 01, UTC + 02, and UTC + 03) was as follows: 12, 354, 4, and 16, respectively. In D2019, the corresponding numbers were 1, 8, 2, and 2 for the same time zones.

**Table 1:** Summary of datasets used in this study (participants and intervals).

Dataset	NP^1^	Interval	Period	Data time	Submission				
					Total	Mean^2^	Median^2^	SD^2^^,^^3^	Range^2^
D2020	386	*interval-highGSI*	7th–20th Sep. 2020	12 am–12 am	128,248	332.25	270	461.13	14–8,081
		*interval-lowGSI*	2nd–15th Nov. 2020		54,270	140.60	109	172.40	11–2,788
D2019	13	*interval-pre*	1st Sep.–30th Nov. 2019	6 am–12 am	31,216	2401.23	1,009	3048.55	276–9,846

1 Number of participants.

2 Overview of data submissions by each participant.

3 Standard deviation.

### 2.3. Analyses

The equivalent continuous SPL and SNR were plotted over the course of the day for all three intervals. To assess the difference of the acoustic features between the three intervals, a *t*-test was conducted, with the grand average acoustic value at each hour as an independent event. Specifically, Welch’s *t*-test was utilized to compare the pre-pandemic and in-pandemic intervals, while a standard independent samples t-test was used to compare the two intervals during the pandemic.

In addition, in D2020, the sound data was reorganized by computing the logarithmic average of SPL and SNR of all submissions within each interval, resulting in a single value of SPL and SNR for each participant in *interval-highGSI* and *interval-lowGSI*, respectively. To further investigate the effect of GSI level (or interval) on SPL and SNR, a repeated analysis of variance (ANOVA) was conducted. All statistical analyses were conducted in Statistical Product and Service Solutions software (SPSS, version 27, IBM Corp., Chicago, IL, United States).

## 3. Results

### 3.1. Comparison of sound exposure before and during the pandemic

#### 3.1.1. Sound pressure level

**Figure 2:**
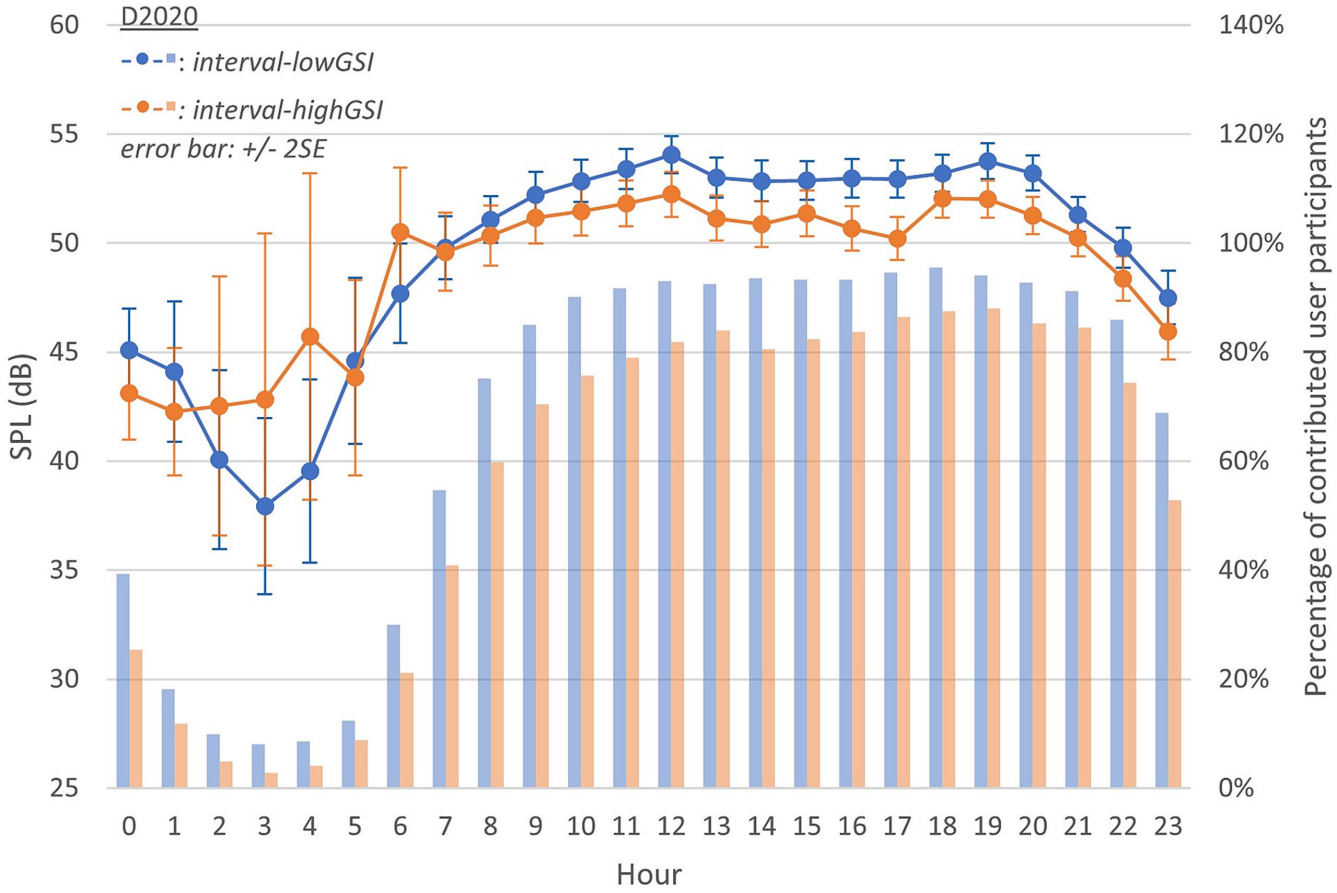
Equivalent continuous sound pressure level (SPL) throughout a day in D2020 (during *interval-highGSI* and *interval-lowGSI* separately; lines follow y-axis on the left, representing acoustic variable values; bars follow y-axis on the right, representing the percentage of participants having data point in sub-conditions; the same interpretation applies to Figure 3).

Figure 2 displays the grand average SPL during *interval-highGSI* and *interval-lowGSI* (represented by lines). In both intervals, the SPL pattern followed a typical course of the day, with a gradual increase in the morning and a decrease at night. It is important to note that the average values need to be considered in combination with the number of participants who submitted data during each hour, shown as bars in Figure 2. In both intervals, the number of participants who submitted data followed a similar daily cycle, with more submissions during the day/evening hours than at night. In addition, as mentioned in Table 1, more participants submitted data during each hour in *interval-lowGSI* than in *interval-highGSI*.

The average SPL was higher in *interval-lowGSI* than in *interval-highGSI* during most hours. However, this order was reversed during the rest hours. It is important to acknowledge that data submissions during nighttime hours were capricious, which could have introduced errors that represent this hour as an outlier.

**Figure 3:**
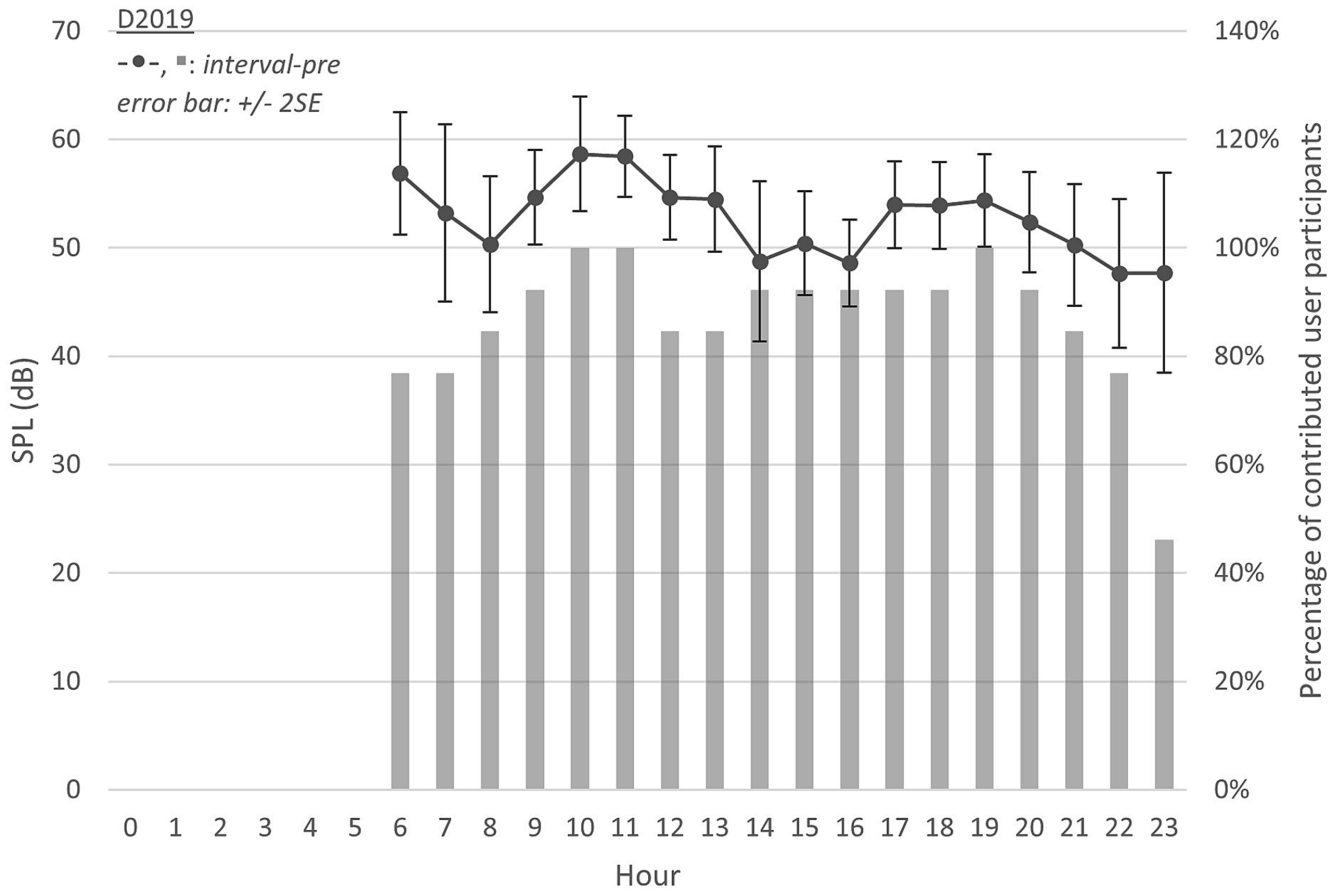
Equivalent continuous SPL throughout 6 am–12 am in D2019 (*interval-pre*).

Figure 3 presents the grand average SPL in *interval-pre* (D2019), which was generally higher than both *interval-highGSI* and *interval-lowGSI* (in D2020), with the exceptions of 14 h, 15 h, and 16 h (see gridline of 50 dB in Figures 2, 3). Nevertheless, this observation is challenging to rationalize due to the limited number of participants in D2019 (N = 13) and the extended observation period (3 months).

Despite the difference in D2020 and D2019, the grand average SPL in each hour across a day could be considered as independent events. A *t*-test was applied between *interval-highGSI*, *interval-lowGSI*, and *interval-pre*. Note that to account for the missing hours in *interval-pre*, data between 0 and 5 h in *interval-highGSI* and *interval-lowGSI* were not included in the test.

In the *t*-test, an alpha level of 0.05 was utilized. The variances in different groups were not homogeneous (F-test, F_1,34_ = 6.655, *p* < 0.05) between *interval-pre* and *interval-lowGSI*. Hence, equal variance was not assumed. Instead of a standard independent samples t-test, a Welch’s *t*-test was conducted between the two intervals, revealing no significant difference [*t* = 0.883, *p* > 0.05, *d* = 0.293 (Cohen’s D)].

Regarding *interval-pre* and *interval-highGSI*, similarly, variances were not homogeneous (F_1,34_ = 14.252, *p* < 0.05). A Welch’s *t*-test revealed a statistically significant difference (*t* = 2.438, *p* < 0.05, *d* = 0.794). The Cohen’s D indicated a strong degree of practical significance (higher d represents bigger effect size), i.e., much lower SPL in *interval-highGSI* compared to in *interval-pre*.

To quantify the difference in SPL between pre-pandemic and in-pandemic, the grand average SPLs observed at different hours in each dataset were used to calculate the following values:

- *L*_eq,24h_: equivalent continuous SPL, calculated as the logarithmic average from 0 to 23 h.
- *L*_eq,6h-23h_: equivalent continuous SPL, calculated as the logarithmic average from 6 to 23 h.

Among the calculated values, *L*_eq,6h-23h_ was included due to D2019 (*interval-pre*) having only data in this range (see Table 2). Although the values were calculated with different hour ranges, a similar trend in *L*_eq,24h_ and *L*_eq,6–23h_ was observed across different intervals. The SPL in *interval-pre* (D2019) was higher than in *interval-lowGSI* (D2020) by 1.74 dB (*L*_eq,6–23h_). Furthermore, the difference between *interval-pre* (D2019) and *interval-highGSI* (D2020) was even greater, reaching 3.18 dB (*L*_eq,6–23h_).

**Table 2:** Summary of the sound pressure level (SPL) in each interval (for D2019, only the *L*_eq,6–23h_ were calculated).

Dataset	Items	*L*_eq,24h_		*L*_eq,6–23h_	
		Mean	(−2SE, +2SE)	Mean	(−2SE, +2SE)
D2020	*interval-lowGSI*	51.18	*(49.34, 53.02)*	52.27	*(51.23, 53.32)*
	*interval-highGSI*	49.84	*(47.06, 52.62)*	50.83	*(49.61, 52.05)*
D2019	*interval-pre*			54.01	*(48.30, 59.71)*

Based on the analyses above, it can be observed that the SPL decreased systematically during the pandemic compared to the pre-pandemic period. The decrease was found to be significant under a stronger governance intervention level (*interval-highGSI*) but not under a milder intervention level (*interval-lowGSI*).

#### 3.1.2. Signal-to-noise ratio

**Figure 4:**
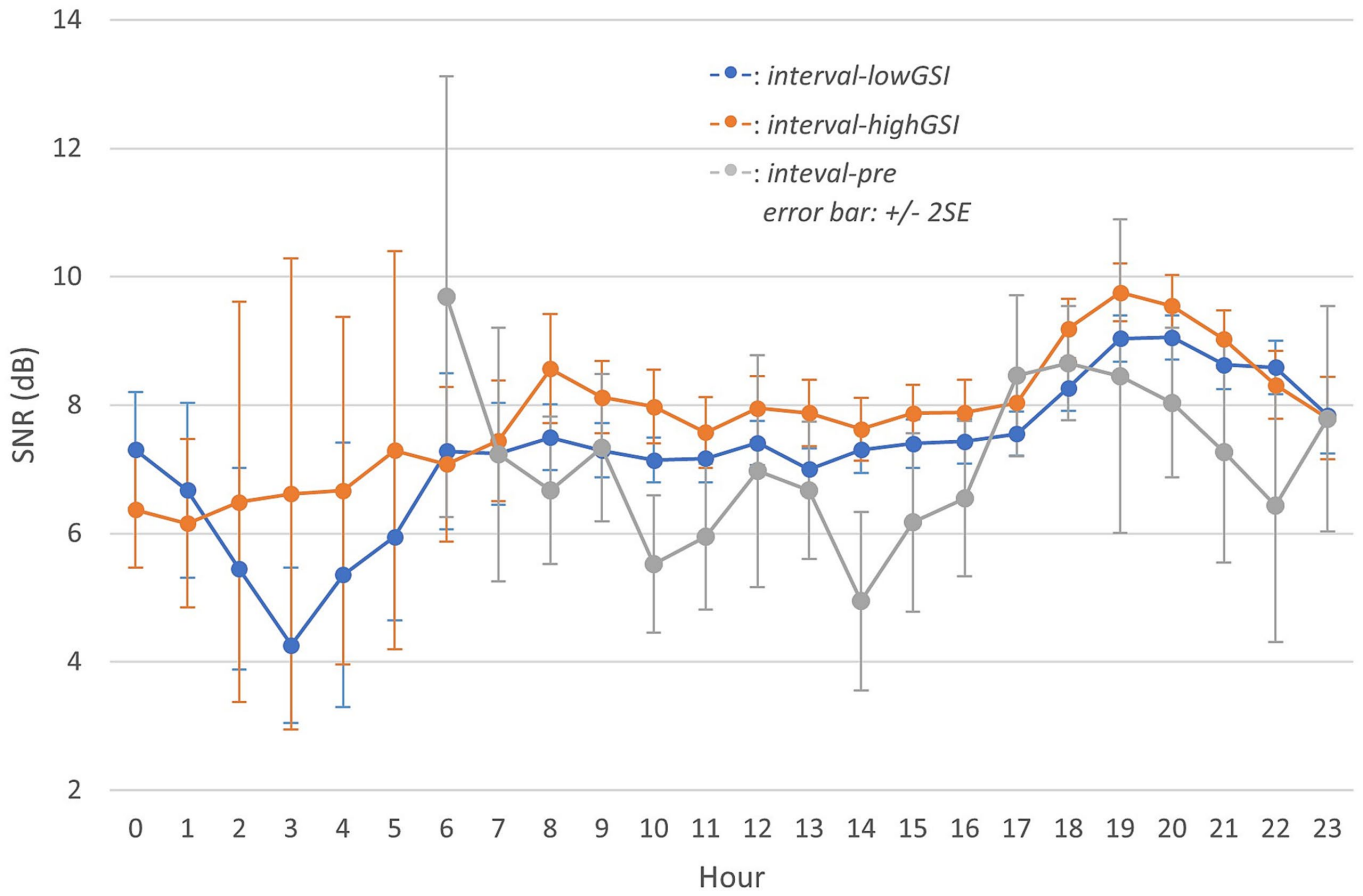
Aggregated signal-to-noise ratio (SNR) throughout a day in different intervals (Information on contributed participants is identical to Figures 2, 3 relatively and hence omitted).

A similar approach was applied to SNR (Figure 4; Table 3). As indicated in “Sound data,” SPL and SNR were acoustic variables estimated from each data submission. Hence, the number of participants submitting data in each specific case was the same as with SPL (Figures 2, 3).

**Table 3:** Summary of the signal-to-noise ratio (SNR) in each interval (for D2019, only the *L*_eq,6–23h_ were calculated).

Dataset	*Items*	*SNR*_eq,24 h_		*SNR*_eq,6–23h_	
		Mean	*(−2SE, +2SE)*	Mean	*(−2SE, +2SE)*
DS20	*interval-lowGSI*	7.39	*(6.67, 8.11)*	7.78	*(7.32, 8.24)*
	*interval-highGSI*	7.91	*(6.72, 9.09)*	8.26	*(7.67, 8.86)*
DS19	*interval-pre*			7.32	*(5.71, 8.92)*

In the pandemic, the grand average SNR remained stable during the day (7–17 h) but increased during the evening hours (18–20 h), and gradually decreased again toward the end of the day (21–23 h). Participants were exposed to higher SNR in *interval-highGSI* than in *interval-lowGSI* at almost all hours, and the grand average SNR during the pre-pandemic period (*interval-pre*) was lower than observed during the pandemic in both *interval-lowGSI* and *interval-highGSI*.

Furthermore, a similar Welch’s *t*-test as in SPL was performed in SNR (alpha level 0.05). Between *interval-pre* and *interval-lowGSI*, variances were not homogeneous (F_1,34_ = 4.337, *p* < 0.05). No significant difference was evident between *interval-pre* and *interval-lowGSI* (*t* = −1.784, *p* > 0.05, *d* = −0.589). As for between *interval-pre* and *interval-highGSI*, again, variances were not homogeneous (F_1,34_ = 3.571, *p* < 0.05). However, a significant difference was observed between *interval-pre* and *interval-highGSI* (*t* = −3.198, *p* < 0.05, *d* = −1.049), with a strong degree of practical significance (Cohen’s D), indicating much higher SNR in *interval-highGSI* compared to in *interval-pre*.

Additionally, SNR_eq,24h_ and SNR_eq,6–23h_ were calculated similarly as for SPL and the result (Table 3) showed that the SNR increased during the pandemic compared to the pre-pandemic, and the increase was significant when under a stronger governance intervention level (*interval-highGSI*) but not when under a milder intervention level (*interval-lowGSI*).

### 3.2. Comparison of sound exposure under different governance intervention levels during the pandemic

“Comparison of sound exposure before and during the pandemic” showed difference in sound exposure between *interval-highGSI* and *interval-lowGSI*. Specifically, *interval-highGSI* had a lower equivalent continuous SPL (Figure 2), and a higher grand average SNR (Figure 4) compared to *interval-lowGSI*. This trend was also observed in *L*_eq,6–23h_ and *SNR*_eq,6–23h_, with differences at −1.44 dB (SPL) and 0.48 dB (SNR).

To further investigate these differences, a *t*-test was conducted between *interval-highGSI* and *interval-lowGSI* in SPL and SNR. In SPL, variances were homogeneous (F_1,34_ = 2.463, *p* > 0.05). Hence, equal variance was assumed. A significant difference was evident between the two intervals [t(34) = 2.195, *p* < 0.05, *d* = 0.732]. In SNR, variances were also homogeneous (F_1,34_ = 0.017, *p* > 0.05). A significant difference was also found [t(34) = −2.008, *p* < 0.05, *d* = 0.669]. The t-test result indicated significantly lower SPL and higher SNR when under a stronger governance intervention level (*interval-highGSI*).

**Figure 5:**
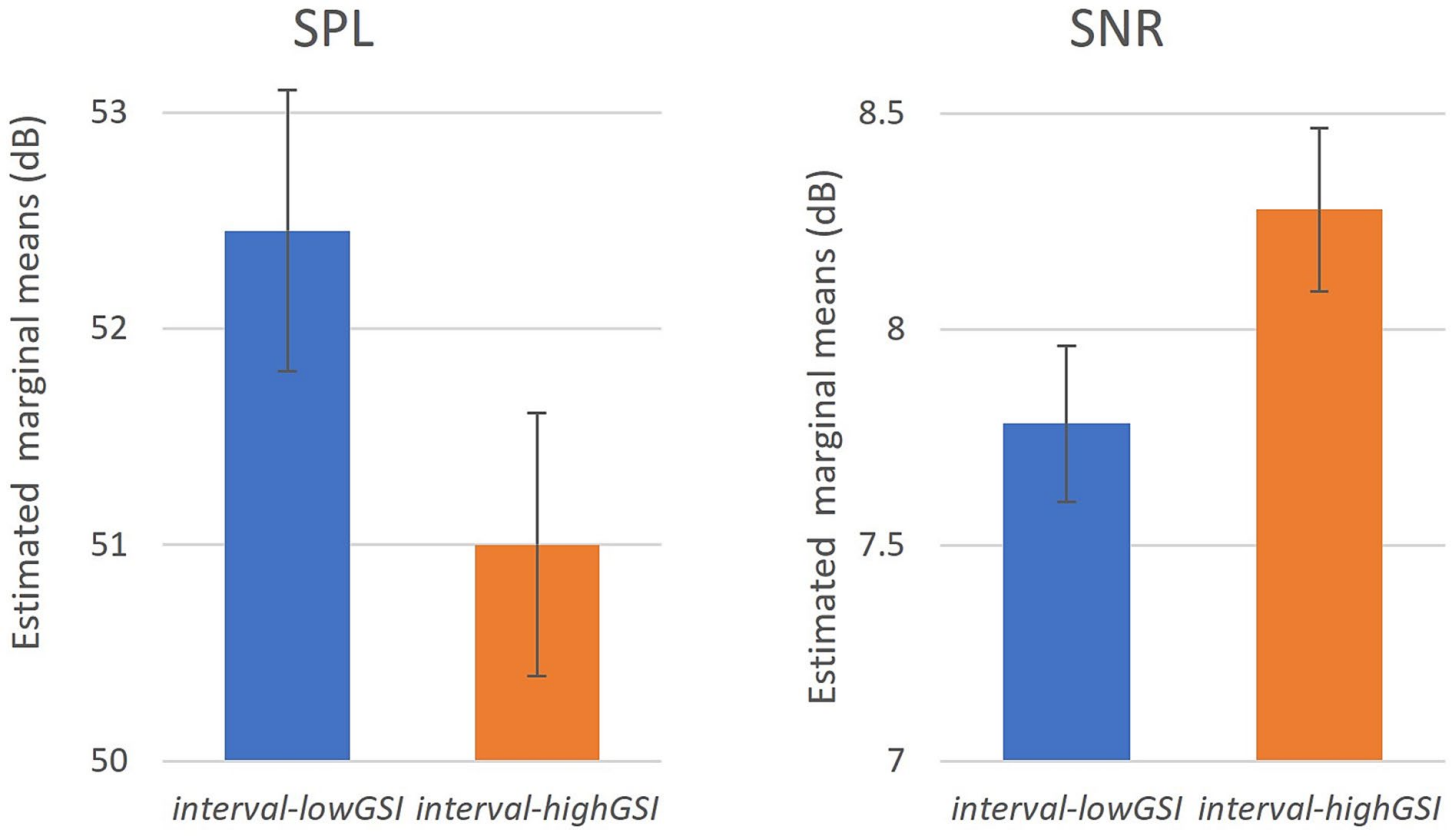
The effect of intervals on SPL and SNR (error bar: 95% CI).

The above analyses were administered on the grand average value at each hour (i.e., at the group average level). Since in D2020, every participant (N = 396) submitted data in both *interval-highGSI* and *interval-lowGSI*, it is of interest to investigate, on an individual level, if the sound exposure was different between the two intervals. As described in “Analyses,” data were reorganized as if each participant reported a single value of SPL and SNR for each interval in D2020. A one-way ANOVA test was conducted using the interval as a fixed factor and acoustic variables as a dependent variable. The results (Table 4; Figure 5) showed that the interval was a significant factor in both SPL and SNR, further supporting the observation that stronger governance intervention levels lead to lower sound exposure and improved sound quality.

**Table 4:** Summary of ANOVA results on acoustic dependents (fixed factor: interval).

Fixed factor: interval		*F*_1,772_	*p*	*R*^2^	Deviation*
dependent	SPL	10.782	0.001	0.14	−1.45 dB
	SNR	12.924	0.000	0.17	0.50 dB

*Average deviation calculated in “interval-highGSI – interval-lowGSI” manner.

## 4. Discussion

### 4.1. General overview

This study conducted continuous observations on a substantial number of HA users (N = 386), using acoustic data obtained from a modern HA device. The study explored two acoustic variables: sound pressure level (SPL) and signal-to-noise ratio (SNR). Two observation periods of 14 days each were selected under varying governance intervention levels, and a separate time interval before the COVID-19 pandemic was chosen as the baseline (with a different set of participants). The result showed that during the pandemic, a decrease in equivalent continuous SPL and an increase in SNR could be observed compared to the pre-pandemic interval (*interval-pre*), particularly when under a stronger governance intervention level (*interval-highGSI*). Furthermore, significant differences in both SPL and SNR were observed during the pandemic across different governance intervention levels, both at the group and individual levels.

### 4.2. Everyday sound exposure for hearing aid users

Previous research (9) examined daily noise exposure in the United States and suggested that median overall daily average levels were 79 and 77 dB (*L*_eq A,8,equiv_ for men and women, respectively) with average levels exceeding recommended limits for 70% of the time. Despite the occupational differences, a Swedish study (39) reported that most daily and weekly noise exposure for workers exceeded the recommended 70 dBA limit for HL prevention. A repeated measures study in Augsburg, Germany (40) identified a wide range of individual daytime noise exposure, with the highest level measured in traffic during bicycling (mean 69 dBA; range 49–97 dBA) and the lowest levels during resting at home (54 dBA; 37–94 dBA). However, these studies did not differentiate between NH and HI individuals.

People with HL face the urge to interact with sounds from the surroundings. The exploration of their daily sound exposure has gained attention in recent years. Doyle et al. (41) followed up sound exposure for HA users across 319 days (mean). Their result implied a mean sound level of 52.38 dB over a day. Furthermore, Christensen et al. (28) performed quantitively sound data collection and found that HA users spent more time in *quiet* and *speech* environments instead of *noise* and *speech-in-noise* environments (i.e., louder and more complex). HL listeners seemed to expose in quieter and less complex environments, which suggested the difference between NH and HL in lifestyles and behaviors. In this study, the observed SPL (e.g., *L*_eq,6–23h_: 50.83–54.01 dB, details see Table 2) values were in line with previous findings, indicating exposure to quieter environments compared to studies that did not specifically focus on HI individuals. Previous research (5) suggested that HI adults, particularly in older adults, experience more emotional distress and less social engagement. While the causality between aging, adverse health outcomes, and less social engagement remains unclear, our findings show that HA users were exposed to a quieter environment, which may contribute to decreased social engagement.

In addition to SPL, SNR plays a crucial role in daily communication for HA users. A previous study (42) have shown a large range of SNR in real-life scenarios, and strikingly few negative or even close to 0 dB SNR. They further reported that for speech-in-babble noise, the average SNR was approximately 5 dB. Similarly, Wu et al. (43) found that 62.9% of real-life recording samples had SNR between 2 to 14 dB and only 7.5% samples had SNR below 0 dB. While audiological studies in laboratory settings may utilize negative SNR [e.g., (44)], it is more common to observe positive SNRs in real-life scenarios. To this end, the observations in this study (Table 3) is in line with literature focusing on HI persons.

The obtained SPL and SNR values in this study are consistent with existing literature, thereby enhancing the validity of our findings. Additionally, our results confirm the differences in lifestyle and behavior between NH and HI individuals. However, the COVID-19 pandemic has affected all residents, and its impact on sound exposure for HI individuals is further discussed in the following section.

### 4.3. The impact of COVID-19 pandemic

#### 4.3.1. Between pre-pandemic and in-pandemic

The impact of governance intervention during the pandemic on sound exposure was multidimensional. One direct impact was that the older adults (who largely overlap with HA users) were discouraged from participating in broader society. Restricted “social bubble” concept and other interventions (19) were used, resulting in withheld society activities such as limited use of shops, restaurants, and public areas in general. Such that the governance interventions led to reduced production of societal ambient sound.

Several studies have investigated the impact of the COVID-19 pandemic on sound exposure. Smith et al. (45) identified a normalized equivalent average 8-h exposures (*L*_EX8h_) changing between the intervention period (with COVID-19 related measures) and baseline period (before the first known COVID-19 death in the US), showing a decrease of 2.6 ± 0.05 dBA. Aletta et al. (46) reported an average reduction of 5.4 dB (L_Aeq_) but varying across different observation sites in London during the pandemic. Rumpler et al. (19) reported that the urban noise level in central Stockholm was reduced by 2–4 dBA (varies at different times of the day), following the government intervention in the “first wave.” However, in their follow-up study (20), such impact was smaller in urban noise level reduction in the “second wave.” Steele and Guastavino (47) revealed that the sound level decreased on the order of 6–7 dB(A) during lockdown, followed by a gradual increased with the relaxation of confinement measures.

In this study, D2019 (*interval-pre*, N = 13) was introduced as a baseline to broadly estimate the impact of the COVID-19 pandemic. Its reliability might not be ideal due to a different and much smaller population compared to D2020. It nevertheless showcased the average sound exposure of HA users between September to November 2019 before the COVID-19 crisis. The COVID-19 pandemic resulted in a decrease in personal ambient sound level for HA users, ranging from 1.74–3.18 dB (∆*L*_eq,6–23h_), which aligns with the range reported in the aforementioned studies. This indicates that despite the differences in lifestyle and behavior between NH and HI (i.e., HL exposed in quieter and less complex environments), both groups were equally impacted by the governance interventions during the pandemic in terms of sound exposure.

In addition to the reduction in SPL, an increase in SNR of 0.46–0.94 dB (∆*SNR*_eq,6h-23h_) was observed, which may potentially explain the better-perceived sound quality reported in Redel-Macías et al. (30). However, a previous laboratory study (48) pointed out that a 3 dB change in SNR is necessary to perceive a just-meaningful difference. Therefore, the increase in SNR observed in this study may not be substantial enough to introduce a just-meaningful difference, but it could contribute to a better-perceived sound quality. Nevertheless, one should avoid overinterpreting just-meaningful differences which require an immediate change in sound. This study focused on relatively long-term sound exposure, which could justify the size of the observed increase in SNR.

#### 4.3.2. Between a milder and a stronger governance intervention level

Between *interval-lowGSI* and *interval-highGSI*, significant differences were observed in both SPL and SNR. Under a stronger intervention level, there was a significant decrease in SPL and an increase in SNR, primarily attribute to the reduction in ambient background sound resulting from the pause of societal activities and increased time spent at home (see Appendix). These findings align with recent studies (20, 30, 47) that have found increased overall level as the COVID-19 restrictions are relaxed. However, it is challenging to determine an effect size from the literature.

Regarding SNR, a naïve assumption is that if a person maintained the same level of social engagement between *interval-lowGSI* and *interval-highGSI*, while let alone the society background sound level changes, the SNR increase should correspond to the decrease in ambient background sound. It is possible that the slight increase in *interval-highGSI* suggest that the reduced personal social interaction was not as severe as the ambient background sound reduction resulting from the governance interventions. However, other factors, such as increased use of alternative sources like TV and music, may have contributed to this result. Nevertheless, heavily limited personal social interaction may simply discourage HA users as well.

However, during the evening hours (Figures 2–4; 20–23 h) across all three intervals, the differences in SPL and SNR were limited. This is likely because these hours are typically spent with family members, which might have been less affected by the COVID-19 restrictions and therefore contributed less to the overall changes in sound exposure.

### 4.4. Prospects

This study highlights the importance of considering SPL and SNR during hearing aid (HA) trials to enhance ecological validity. Despite the pandemic leading to reduced ambient noise and improved SNR, HA users reported worsened anxiety, depression, and hearing difficulty (49). In fact, noise complaints increased during the pandemic in general (50). Research on soundscape (51–53) indicated that “the quieter, the better” strategy might not be the best option to optimize the experience of urban soundscapes.

On one hand, HA trials should incorporate realistic test conditions to better align HA performance with users’ daily experiences. On the other hand, hearing healthcare services, including consultation, prescription, and device selection, should not only address present communication challenges but also aim to support individuals with hearing impairment in various situations, ultimately improving their overall well-being. Furthermore, the impact of significant societal changes like the COVID-19 pandemic should be considered in clinical protocols. Objective data such as sound exposure and HA usage should be taken into account during clinical diagnoses and consultations to better address hearing dissatisfaction beyond users’ subjective descriptions. Enhancing the hearing experience and hearing healthcare in such circumstances presents challenges for stakeholders but also offers opportunities for innovation and growth.

It is important to recognize that the population of individuals with hearing loss extends beyond HA users. The participants in this study may have been more active, technologically adept, or even extroverted compared to the average population with hearing loss, given their use of hearing devices and mobile apps. The concerning findings of low sound exposure in this study, indicating limited social engagement, may be even more prevalent among individuals with hearing loss in general. Therefore, hearing healthcare faces the challenge of encouraging individuals with hearing loss to expand their social interactions by providing optimal and personalized support. In turn, society must raise awareness of the benefits for individuals with hearing loss and focus on preventive measures. It requires a collaborative effort between society, hearing healthcare professionals, and individuals to address these issues effectively (54).

### 4.5. Limitations

Despite the insights observed in this study, several limitations should be considered. Firstly, the participants were spanned across Europe without clear geographic information, potentially leading to the variations in local restrictions and compliance despite the general agreed overall GSI trend. Using time zone as a proxy of geography makes it possible to include /exclude participants from some specific countries, such as South Africa, in this study. However, we genuinely believe the included European countries collectively adhered to a common framework during the COVID-19 pandemic, as well as South Africa. Moreover, the majority of participants are expected to come from the included European countries. Subsequent investigations may benefit from focusing on a single country or region to gather more comprehensive data, while ensuring an adequate sample size. Secondly, a minimum of 10 submissions in each interval in D2020 was required but was not examined due to the priority of inclusivity. Additionally, while the governance intervention level (GSI) was assumed to be the main difference between the two intervals in D2020, other factors such as the time of the year might also play a role. Previous research (55, 56) has identified the effect of seasonality (at a 6-month interval) on community noise annoyance, but the smaller interval gap in this study suggested a general fluctuation rather than a significant difference due to the time of the year between low and high GSI. Furthermore, it should be noted that the data in this study was obtained using a single module and brand of device, designed to comply with relevant regulations and standards. While we have confidence in the obtained data, it is important to acknowledge that other modules or brands may show different results. Exploring the use of multiple modules and brands in future studies would provide valuable insights into potential variations in sound data collection. Although this is beyond the scope of this paper, we believe that further exploration in this area will contribute to a deeper understanding of the subject matter.

## 5. Conclusion

Based on the findings from this study, it can be concluded that the COVID-19 pandemic had a significant impact on the sound exposure of HA users in Europe. The study included 386 participants and revealed that the equivalent continuous SPL was generally lower, and the SNR was higher during the pandemic. Moreover, the sound exposure during the pandemic was compared between two intervals during the pandemic, and a stronger governance intervention level was found to resulted in a significantly lower SPL and higher SNR. These results are in agreement with recent research on urban noise monitoring during the COVID-19 pandemic and previous research on noise exposure of HA users. Overall, this study provides insights into the sound exposure of HA users during the pandemic.

## Data availability statement

The datasets presented in this article are not readily available because participants’ consent forms (privacy policy) grant Demant Group permission to utilize their data for research purposes while adhering to strict guidelines regarding data sharing with external entities. Requests to access the datasets should be directed to KS, knsu@eriksholm.com.

## Ethics statement

Ethical review and approval were not required for the study on human participants in accordance with the local legislation and institutional requirements. Written informed consent for participation was not required for this study in accordance with the national legislation and the institutional requirements.

## Author contributions

KS and T-IS designed the study and organized the database. KS performed the statistical analysis and wrote the manuscript. AP, LB, DW, JC, and NP supervised the findings and revised the final manuscript. All authors contributed to the article, read, and approved the submitted version.

## Funding

This research was supported by Innovation Fund Denmark and William Demant Foundation (no. 9066-00013B). These organizations are gratefully acknowledged.

## Footnotes

^1^www.h2020evotion.eu

^2^https://www.nvk.dk/forsker/naar-du-anmelder/hvilke-projekter-skal-jeg-anmelde

^3^https://www.oticon.global/hearing-aid-users/support/information/downloads
