# Supplementary material for: Daily sound exposure of hearing aids users during COVID-19 pandemic in Europe
Source: Front Public Health. 2023 Oct 13;11:1091706. doi: 10.3389/fpubh.2023.1091706 (PMC10613490; doi:10.3389/fpubh.2023.1091706)
Supplement: Supplementary file 1 [file Table_1.pdf]

Appendix

Figure 1 illustrates the GSI for (some) European countries, which could be subdivided by focusing on different aspects. The following figure further shows the evolvement of such aspects, acquired from the same data resource. As illustrated in Figure A, relative to *interval-lowGIS*, participants spent more time at home and less time in visiting parks in *interval-highGSI*. As expected, the public gathering rules were more restricted following the stronger government intervention in *interval-highGSI*. However, in terms of visiting the workplace, the difference therein seemed not significant.

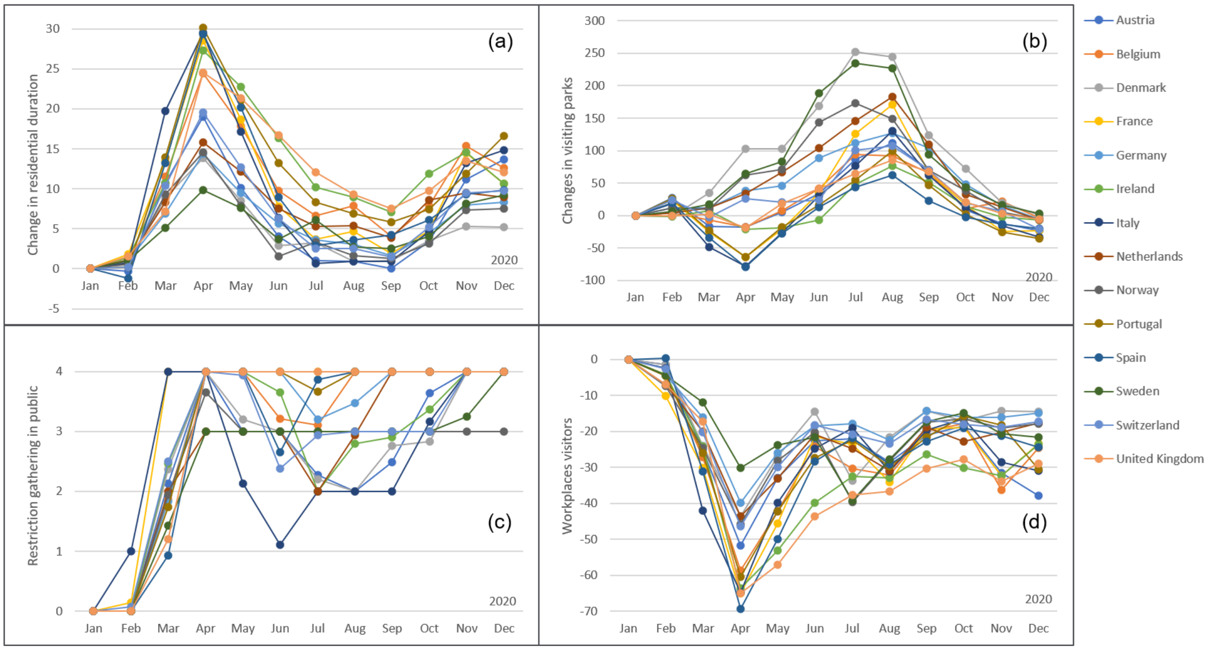

Figure A – Month-based multi-dimensional behavior estimator in European countries across 2020.

a: residential duration, represents changes in the duration of time spent in places of residence (in percentage) relative to a baseline day (0);

b: park visit, represents changes in visitors to parks (in percentage) relative to a baseline day (0);

c: public gathering restrictions, 0 represents no restrictions while 4 represents restrictions on gathering of fewer than 10 people;

d: workplace visit, represents changes in visitors to workplaces (in percentage) relative to a baseline day.

(*: Baseline day is the median value from a 5-week period observation

**: Resource: Hale et al., 2021. For the day-based government stringency index, we refer readers to the original resource.).
